# Low nutrient intake among adult women and patients with severe tuberculosis disease in Uganda: a cross-sectional study

**DOI:** 10.1186/1471-2458-12-1050

**Published:** 2012-12-05

**Authors:** Ezekiel Mupere, Isabel M Parraga, Daniel J Tisch, Harriet K Mayanja, Christopher C Whalen

**Affiliations:** 1 Department of Paediatrics & Child Health School of Medicine College of Health Sciences, Makerere University Kampala, Kampala, Uganda; 2 Department of Nutrition, Case Western Reserve University Cleveland Ohio, Cleveland, USA; 3 Department of Epidemiology & Biostatistics, Case Western Reserve University Cleveland Ohio, Cleveland, USA; 4 Department of Internal Medicine School of Medicine College of Health Sciences, Makerere University, Kampala, Uganda; 5 Department of Epidemiology & Biostatistics, College of Public Health, University of Georgia, Athens, USA

**Keywords:** Tuberculosis disease severity, Gender, Dietary intake, Body wasting, HIV

## Abstract

**Background:**

Information regarding dietary nutrient intake during tuberculosis disease is lacking. We established the relationship between disease severity or wasting during pulmonary tuberculosis and nutrient intake.

**Methods:**

In a cross-sectional study of 131 adults with or without pulmonary tuberculosis were screened for human immune-deficiency virus (HIV), wasting, disease severity using 13 item validated clinical TBscore, and 24-hour dietary intake recall.

**Results:**

Of the 131 participants, 61 were males and 70 females. Overall men and women had similar age. In average 24-hour nutrient intake, the following nutrients: energy, protein, total fat, carbohydrate, calcium, vitamin A, and folate were low among patients with severe tuberculosis disease. Patients with moderate-to-severe clinical TBscore had lower average energy intake than patients with mild TBscores (6.11 vs. 9.27 MJ, respectively) (p<0.05). The average 24-hour nutrient intakes between wasted and non-wasted tuberculosis patients were comparable. Nutrient intake among men was higher when compared to women regardless of wasting and severity of tuberculosis. Among those with wasting, men had higher average energy intake than women (8.87 vs. 5.81 MJ, respectively) (p<0.05). Among patients with mild disease, men had higher average energy intake than women with mild disease (12.83 vs. 7.49 kcal, respectively) (p<0.001).

**Conclusions:**

Findings suggest that severity of pulmonary tuberculosis and female gender had reduced nutrient intake. Early tuberculosis diagnosis and nutritional support may be important in management of tuberculosis patients.

## Background

Sub-Saharan Africa bears the highest burden of tuberculosis patients with human immunodeficiency virus (HIV) [1]. An estimated 1.37 million new cases of tuberculosis with HIV co-infection occurred in 2007; 79% of which were from sub-Saharan Africa. Co-infection with tuberculosis and HIV poses an extra burden to the pathophysiology of wasting, exacerbating the wasting process seen in tuberculosis or HIV infection alone [2,3]. Moreover, co-infection and malnutrition have deleterious interactions. Co-infected patients with malnutrition have an increased risk of morbidity and mortality [2,4,5] and tuberculosis is the leading cause of death in co-infected patients in tuberculosis endemic countries, including those with free access to antiretroviral therapy [6].

Co-infection may lead to poor appetite with decreased nutrient intake, which may interact with altered metabolism as part of the immune and inflammatory responses [7] leading to exacerbation of the existing body wasting. Moreover, poor nutritional status is associated with risk of tuberculosis relapse [8] and mortality. The goal of nutritional assessment and nutritional support is to intervene early and to preserve lean tissue mass compartment, from further wasting because disproportionate loss of lean tissue mass is associated with morbidity and mortality [9]. Lean tissue mass is a marker of body wasting and malnutrition; it is a consequence of negative imbalance between energy (and protein) needs and dietary intake that occurs for more than a few days.

Assessment of dietary nutrient intake is essential in nutritional management. Despite the high burden of malnutrition and the high burden of co-infection with associated body wasting, assessment of nutrition intake is often neglected in clinical practice and in national tuberculosis programs. Thus, the effects of tuberculosis on nutrient intake have not been well described. The present cross-sectional study was conducted to establish the relationship between nutrient intake and body wasting and between nutrient intake and severity of tuberculosis disease at the time of tuberculosis diagnosis.

## Methods

In a cross-sectional study, 137 ambulatory participants 18 years or older residing in Kampala district or 20 km from the study site if residence was outside Kampala in Uganda were contacted to participate in the study. Participants with prior tuberculosis treatment and those who declined written consent were excluded. The study was conducted at the National Tuberculosis and Leprosy Program (NTLP) Clinic of Mulago tertiary teaching hospital complex between November 2007 and May 2008. The tuberculosis patients were recruited at Mulago NTLP Clinic; HIV sero-positive participants without tuberculosis at the Infectious Disease Institute Clinic (IDI) located 300 meters away from the Mulago NTLP Clinic; and HIV sero-negative participants without tuberculosis from the community where enrolled tuberculosis patients resided. The community individuals were selected through random pick of consenting adults from the immediate neighborhood households to those for tuberculosis patients who had presented at the hospital.

The institutional review boards at Case Western Reserve University and Joint Clinical Research Center approved the study, with final approval by the Uganda National Council for Science and Technology. All participants provided written informed consent to the study.

All subjects in the study were given appropriate pre- and post-test HIV counseling and AIDS education. HIV-1 infection was diagnosed on the basis of a positive enzyme-linked immunosorbent assay for HIV-1 antibodies (Recombigen; Cambridge Biotech, Cambridge, MA). At enrollment, basic demographic information and a medical history were collected, and a standardized physical examination was conducted by a medical officer. Active pulmonary tuberculosis was confirmed by sputum smear microscopy and culture. Patients with active tuberculosis were treated with standard four-drug chemotherapy for tuberculosis per guidelines of the Ugandan Ministry of Health. HIV sero-positive patients eligible for antiretroviral therapy were started on treatment and cotrimoxazole prophylaxis at the IDI clinic.

Nutrient intake assessment was made with a single 24-hour dietary recall using a pre-tested questionnaire. Assessment was conducted by four trained study staff nurses and supervised by a nutritionist using local food photographs, portion-size images, and volumetric vessels to increase the accuracy of the recall from the previous 24 hours. The nutritive value of ingredients was computed using the East African food composition table database whose database was imported into the NutriSurvey software ( http://www.nutrisurvey.de) to perform computations. When the East African food composition table was found deficient in certain food items, the United States Department of Agriculture database and the African composition table were used.

We defined body wasting of participants using body mass index (BMI) and height-normalized indices (adjusted for height) for body composition of lean mass index (LMI) and fat mass index (FMI). BMI can be partitioned into height-normalized LMI and FMI as explained as elsewhere [10-12]. Height-normalized LMI and FMI eliminate differences in fat and fat-free mass associated with height [12,13]. The LMI and FMI have the advantages of compensating for differences in height and age [14]; the use of the LMI and FMI also eliminates some of the differences between population groups. We defined body wasting according to sex. For men, wasting was present when the LMI was below 16.7 (kg/m^2^) and/or the FMI was below 1.8 (kg/m^2^). For women, wasting was present with the LMI below 14.6 (kg/m^2^) and/or FMI below 3.9 (kg/m^2^). These cutoffs correlate with the BMI according to the WHO cutoff for malnutrition [10,11,15] among adults.

Anthropometric measurements included height and weight. BMI was computed using the relationship of weight in kilograms divided by height in meters squared (kg/m^2^). Weight was taken using Hanson digital electronic scales to the nearest 100g. Height was measured to the nearest cm of standing height using a stadiometer. All anthropometric measurement values were means of duplicates.

The single-frequency bioelectrical impedance analyzer (BIA Detroit, MI, RJL Systems) performing at 50 kHz and 800 mA was used for BIA measures with detecting electrodes placed on the wrist and ankle and signal introduction electrodes placed on the first joint of the middle finger and behind the middle toe. Before performing measurements on each subject, the BIA instrument was calibrated using the manufacturer’s recalibration device. Measurements were taken at rest in participants with voided bladder, ambient temperature, and had not exercised or taken alcohol. The resistance and reactance were based on measures of a series circuit [16]. BIA measurements were performed in duplicate for each subject. Lean tissue mass was calculated from BIA measurements using equations that were previously cross-validated in a sample of patients (white, black and Hispanic) with and without HIV infection [16] and have been applied elsewhere in African studies [17-19]. Fat mass was calculated as body weight minus lean tissue mass.

We classified tuberculosis patients into two categories of clinical disease severity using the clinical TBscore [20]. The clinical TBscore is a low-cost tool that has been developed and validated to assess severity of clinical tuberculosis disease in resource limited settings [20]. The score has a maximum of 13 clinical variables each scoring 1 point as follows: self-reported symptoms of cough, hemoptysis, dyspnea, chest pain, and night sweating; anemic conjunctivae, tachycardia, positive finding at lung auscultation, axillary temperature >37.0^0^C, BMI <18, BMI <16, middle upper arm circumference (MUAC) <220 mm, and MUAC <200 mm. Two clinical severity categories were generated using the following cutoffs as reported elsewhere [20]: mild 0 – 5 and moderate-to-severe > 5. Higher scores are associated with severe disease. HIV sero-negative participants without tuberculosis were classified as no disease category.

### Analysis

All study participants in the analysis were categorized into 1) 4 mutually independents groups: HIV sero-positive patients with and without tuberculosis disease, HIV sero-negative patients with and without tuberculosis, 2) wasted and non-wasted using cutoff of low BMI and low LMI, and 3) mild and moderate-to-severe TB disease using the clinical TBscore [20]. Measures of central tendency and variability were compared between women and men across 4 mutually exclusive groups, between patients with and patients without wasting, and between patients with mild and with moderate-to-severe tuberculosis disease using Wilcoxon-Mann Whitney test for average weight, height, BMI, LMI, FMI, and nutrient intake parameters due to lack of normality. A p-value of <0.05 was considered significant in all analyses. A sample size of 14 participants was computed per group to detect 5 mega joules (MJ) difference in energy intake between wasted and participants without wasting or between patients with mild and patients with moderate-severe disease assuming type I error of 5%, 80% power, and normal distribution with standard deviation of 4.5. All analyses were performed using SAS version 9.2 (Cary software, North Carolina SAS Institute Inc 2004).

## Results

### Characteristics of study population

Of the 137 potential participants who were contacted, four declined consent, one had prior tuberculosis treatment and one did not complete all the necessary study evaluations. Of the 131 participants who were involved in the analysis, 61 were men and 70 were women. Further of the 131 who were analyzed, 31 were HIV sero-positive with tuberculosis, 32 were HIV sero-negative with tuberculosis, 38 were HIV seropositive without tuberculosis, and 30 were HIV sero-negative without tuberculosis. Overall men and women in the study population had similar age regardless of HIV status except among HIV positive individuals without tuberculosis; men were significantly older than women 34.7 versus 29.7, respectively. Among HIV sero-positive and HIV sero-negative patients with tuberculosis, men and women had comparable BMI whereas among HIV sero-positive and HIV sero-negative individuals without tuberculosis, women had significantly higher BMI compared to men (Table 1). Men had a higher height-normalized LMI and a lower height-normalized FMI compared to women regardless of tuberculosis and HIV status.

**Table 1:** Body composition and severity of disease among HIV positive and HIV negative individuals with/without tuberculosis

**Characteristic [mean, (SD)]**^**c**^	**HIV positive with TB (n=31)**		**HIV negative with TB (n=32)**		**HIV positive without TB (n=38)**		**HIV negative without TB (n=30)**	
	**Men (n=10)**	**Women (n=21)**	**Men (n=18)**	**Women (n=14)**	**Men (n=17)**	**Women (n=21)**	**Men (n=16)**	**Women (n=14)**
Age in yrs	30.9 (4.6)	29.2 (5.9)	26.0 (7.3)	26.3 (4.6)	34.7 (6.5)	29.7 (8.4)^b^	22.4 (3.2)	24.3 (5.4)
BMI kg/m^2^	18.4 (1.7)	18.6 (3.0)	18.2 (2.0)	20.3 (4.3)	21.2 (2.2)	24.2 (4.6)^b^	21.6 (2.3)	23.7 (2.8)^b^
LMI in kg/m^2^	16.6 (1.3)	15.4 (0.9)^b^	16.6 (1.5)	16.1 (0.9)	18.3 (1.2)	16.6 (1.2)^a^	18.6 (1.4)	16.6 (1.1)^a^
FMI in kg/m2	1.8 (0.6)	3.3 (2.1)	1.6 (0.8)	4.6 (3.6)^a^	3.2 (1.2)	7.6 (3.7)^a^	2.9 (1.0)	7.1 (2.1)^a^
Severity TBscore	6.5 (1.5)	5.6 (2.6)	6.8 (2.3)	5.9 (2.4)	-	-	-	-

^a^p-value <0.001, ^b^p-value <0.05. ^c^Characteristic values are means ± standard deviation (SD)BMI = body mass index, LMI = lean mass index, FMI = fat mass index, SD = standard deviation.

### Body composition in the study population

Tuberculosis but not HIV infection was associated with reductions in body mass compartment. BMI, LMI, and FMI, for both men and women, were reduced due to tuberculosis among both HIV seropositive and seronegative subjects (Table 1). For example, among HIV seropositive men, LMI in patients with tuberculosis was 16.6 ± 1.3 kg/m^2^ compared with 18.3 ± 1.2 kg/m^2^ in patients without tuberculosis (P = 0.006). Similarly, for HIV seropositive women, LMI in tuberculosis patients was 15.4 ± 0.9 kg/m^2^ compared to 16.6 ± 1.2 kg/m^2^ in patients without tuberculosis (p = 0.002). Among HIV seropositive men, FMI in patients with tuberculosis was 1.8 ± 0.6 kg/m^2^ compared with 3.2 ± 1.2 kg/m^2^ in patients without tuberculosis (P = 0.003). Similarly for HIV seropositive women, FMI in tuberculosis patients was 3.3 ± 2.1 kg/m^2^ compared to 7.6 ± 3.7 kg/m^2^ in patients without tuberculosis (p < 0.001). The relationships were similar when comparing HIV seronegative persons with and without tuberculosis according to sex. HIV infection did not affect severity of tuberculosis disease. The clinical tuberculosis severity score was comparable between HIV seropositive and HIV seronegative patients with tuberculosis, and was comparable between men and women regardless of HIV status among patients with tuberculosis (Table 1). For example, the score was 6.5 ± 1.5 for men compared to 5.6 ± 2.6 for women (p = 0.414).

### Differences in nutrient intake by tuberculosis disease and HIV status

There were no differences in average 24-hour nutrient intake by Tuberculosis disease and HIV status. Average 24-hour nutrient intake for energy, protein, total fat, carbohydrate, calcium, magnesium, zinc, iron, vitamin A, and folate were comparable among participants with or without tuberculosis and among tuberculosis patients with or without HIV regardless of gender (Table 2). For example, among HIV seropositive men, energy intake in patients with tuberculosis was 8.49 ± 4.65 MJ compared with 8.18 ± 2.93 MJ in patients without tuberculosis (P>0.05). Similarly for HIV seropositive women, energy intake in tuberculosis patients was 6.02 ± 3.85 MJ compared to 6.91 ± 4.63 MJ in patients without tuberculosis (p > 0.05). Of note, however, the average 24-hour intake for most nutrients was lower for women compared to men but no statistical significant differences.

**Table 2:** Average 24-hour nutrient intake among HIV-seropositive and HIV-seronegative patients with or without pulmonary tuberculosis

**Characteristic [mean, (SD)]**^**c**^	**Men (n=61)**				**Women (n=70)**			
	**HIV+ (n=27)**		**HIV- (n=34)**		**HIV+ (n=42)**		**HIV- (28)**	
	**TB+ (n=10)**	**TB- (n=17)**	**TB+ (n=18)**	**TB- (n=16)**	**TB+ (n=21)**	**TB- (n=21)**	**TB+ (n=14)**	**TB- (n=14)**
Energy (MJ)	8.49 (4.65)	8.18 (2.93)	8.77 (3.48)	9.96 (2.94)	6.02 (3.85)	6.91 (4.63)	6.56 (2.34)	6.69 (2.37)
Protein (g)	60.6 (48.6)	56.9 (47.2)	56.0 (29.5)	60.4 (21.1)	49.5 (44.2)	38.3 (24.8)	42.8 (22.3)	35.9 (16.3)
Total fat (g)	59.5 (60.2)	47.2 (28.5)	56.6 (38.9)	60.7 (25.1)	32.4 (38.4)	34.0 (20.0)	39.8 (24.9)	38.2 (23.3)
Carbohydrate (g)	303 (158)	333 (125)	348 (139)	416 (119)	244 (141)	301 (242)	269 (82)	274 (124)
Calcium (mg)	978 (1532)	415 (414)	297 (319)	417 (454)	558 (1029)	263 (404)	264 (200)	346 (406)
Magnesium (mg)	267 (267)	238 (204)	302 (160)	278 (181)	261 (250)	251 (336)	176 (134)	190 (150)
Zinc (mg)	7.7 (5.9)	7.1 (4.3)	6.9 (3.2)	7.2 (3.3)	5.6 (4.5)	5.7 (4.8)	4.4 (2.1)	3.8 (1.8)
Iron (mg)	11.9 (12.5)	11.1 (7.7)	11.8 (8.2)	12.4 (7.0)	11.5 (12.6)	11.5 (14.2)	9.0 (5.4)	7.0 (5.3)
Vitamin A (RE)	469 (568)	188 (238)	545 (835)	936 (2716)	430 (493)	959 (1794)	779 (745)	285 (314)
Folate (μg)	300 (221)	362 (174)	346 (257)	513 (218)^b^	315 (278)	312 (190)	375 (183)	293 (148)

p-values obtained with Wilcoxon test; ªp-value < 0.001, ^b^p-value < 0.05. ^c^Characteristic values are means ± standard deviation (SD). TB- = no TB disease, TB+ = Tuberculosis disease, HIV+ = HIV infection.

### Differences in nutrient intake by severity of pulmonary tuberculosis disease and wasting status

Patients with severe pulmonary tuberculosis disease but not wasting had reduced 24-hour nutrient intake. The average 24-hour intake for energy, protein, total fat, carbohydrate, calcium, vitamin A, and folate were significantly lower among tuberculosis patients with moderate-to-severe disease compared to patients with mild disease. For example, among patients with moderate-to-severe TBscore average energy intake was 6.11 ± 3.02 MJ compared with 9.27 ± 3.94 MJ among patients with mild TBscore (p<0.05, Table 3). However, there were no significant differences in average 24-hour nutrient intake between tuberculosis patients with reduced lean tissue mass or reduced BMI and patients with normal lean tissue mass or normal BMI (Table 3).

**Table 3:** Average 24-hour nutrient intake stratified by wasting and pulmonary tuberculosis disease severity

**Characteristic [mean, (SD)]**^**c**^	**Lean tissue mass**		**BMI**		**Disease severity**	
	**Not wasted (n=43)**	**Wasted (n=20)**	**Not wasted (n=28)**	**Wasted (n=35)**	**Mild ≤5 (n=24)**	**Moderate/severe >5 (n=39)**
Energy (MJ)	7.03 (3.31)	7.93 (4.48)	7.23 (2.94)	7.38 (4.26)	9.27 (3.94)	6.11 (3.02)^b^
Protein (g)	50.3 (33)	54.5 (44.3)	52.4 (36.8)	51.0 (37.3)	70.5 (46.4)	40 (20.6)^b^
Total fat (g)	39.4 (32.3)	57.8 (54.2)	39.1 (22.8)	50.2 (50.9)	61.6 (50.3)	35.2 (30.6)^b^
Carbohydrate (g)	288 (138)	290 (137)	296 (140)	283 (135)	348 (130)	252 (128)^b^
Calcium (mg)	448 (759)	565 (1125)	529 (895)	450 (885)	772 (1330)	308 (344)^b^
Magnesium (mg)	244 (193)	279 (242)	254 (161)	255 (242)	312 (245)	220 (177)
Zinc (mg)	5.7 (3.4)	6.8 (5.3)	5.5 (2.5)	6.5 (5.0)	7.2 (5.0)	5.3 (3.3)
Iron (mg)	11.2 (9.4)	10.9 (11.4)	10.3 (7.2)	11.7 (11.8)	12.5 (10.9)	10.2 (9.5)
Vitamin A (RE)	559 (608)	519 (807)	588 (708)	514 (650)	751 (798)	421 (555)^b^
Folate (μg)	351 (227)	298 (270)	330 (225)	338 (256)	452 (283)	262 (179)^b^

p-values obtained with Wilcoxon test; ^b^p-value <0.001, ^b^p-value <0.05. ^c^Characteristic values are means ± standard deviation (SD). BMI = body mass index; BMI wasting = <18.5 kg/m^2^ for men and women; Lean tissue mass wasting = Lean tissue mass index <16.7 kg/m^2^ for men and <14.6 kg/m^2^ for women; and moderate-severe tuberculosis disease = TBScore >5 [20].

Patients with severe pulmonary tuberculosis disease had decreased 24-hour nutrient intake regardless of the wasting status. Energy, protein, total fat, carbohydrate, calcium, zinc, iron, vitamin A, and folate for both patients with or without body wasting regardless of the method used for assessing nutritional status, were reduced in magnitude among patients with severe tuberculosis (Table 4). Among patients that had reduced LMI, energy intake in patients with mild disease was 8.19 ± 3.25 MJ compared with 6.02 ± 3.07 MJ in patients with moderate-severe disease (P<0.05). For patients that had reduced BMI, energy intake in patients with mild disease was 10.29 ± 4.85 MJ compared to 6.05 ± 3.28 MJ in patients with severe disease (p<0.05). Among patients that had normal LMI, energy intake in patients with mild disease was 14.65 ± 2.32 MJ compared with 6.24 ± 3.04 MJ in patients with moderate-sever disease (P<0.05). For patients who had normal BMI, energy intake in patients with mild disease was 8.41 ± 2.89 MJ compared to 6.21 ± 2.66 MJ in patients with moderate-severe disease (Table 4). However, there were no significant differences in average 24-hour nutrient intake between wasted patients with moderate-severe disease and non-wasted patients with moderate-severe disease.

**Table 4:** Average 24-hour nutrient intake among wasted and non-wasted patients stratified by tuberculosis disease severity

**Characteristic [mean, (SD)]**^**c**^	**Lean tissue: Not wasted**		**Lean tissue: Wasted**		**BMI: Not wasted**		**BMI: Wasted**	
	**Mild (n=23)**	**Severe (n=20)**	**Mild (n=16)**	**Severe (n=4)**	**Mild (n=13)**	**Severe (n=15)**	**Mild (n=11)**	**Severe (n=24)**
Energy (MJ)	14.65 (2.32)	6.24 (3.04)^b^	8.19 (3.25)	6.02 (3.07)^b^	8.41 (2.89)	6.21 (2.66)	10.29 (4.85)	6.05 (3.28)^b^
Protein (g)	130.2 (36.2)	35.5 (17.7)^b^	58.6 (41.6)	43.1 (22.3)	66.0 (47.9)	40.6 (18.1)	75.9 (50.8)	39.6 (22.5)^b^
Total fat (g)	126.3 (57.3)	40.6 (38.8)^b^	48.6 (38.6)	31.5 (23.7)	46.7 (22.7)	32.5 (21.5)	79.2 (67.6)	36.9 (35.5)
Carbohydrate (g)	430 (133)	255 (117)	332 (127)	250 (138)^b^	339 (139)	259 (135)	359 (125)	248 (126)^b^
Calcium (mg)	1732 (2334)	273 (242)	579 (1024)	333 (404)	676 (1221)	401 (481)	884 (1502)	251 (214)
Magnesium (mg)	626 (320)	192 (116)^b^	249 (178)	239 (209)	267 (190)	243 (138)	365 (299)	205 (199)
Zinc (mg)	16.2 (2.5)	4.4 (2.2)^b^	5.4 (2.9)	5.9 (3.9)	5.7 (2.8)	5.4 (2.4)	9.1 (6.3)	5.3 (3.8)
Iron (mg)	24.3 (20.3)	7.5 (4.9)	10.2 (6.6)	12.0 (11.4)	10.4 (7.3)	10.2 (7.4)	15.0 (14.0)	10.1 (10.7)
Vitamin A (RE)	1094 (1252)	376 (634)^b^	682 (702)	452 (505)	716 (812)	477 (610)	792 (818)	386 (528)^b^
Folate (μg)	487 (429)	251 (209)	445 (260)	270 (159)^b^	420 (254)	251 (167)	489 (322)	270 (189)

p-values obtained with Wilcoxon test; ^a^p-value <0.001, ^b^p-value <0.05. ^c^Characteristic values are means ± standard deviation (SD). BMI = body mass index. BMI wasting = <18.5 kg/m^2^ for men and women; Lean tissue mass wasting = Lean tissue mass index <16.7 kg/m^2^ for men and <14.6 kg/m^2^ for women; moderate-severe tuberculosis disease = TBScore >5 [20].

### Differences in nutrient intake by gender

The average 24-hour nutrient intake among men was higher when compared to women regardless of the method used to assess body wasting status and the severity of pulmonary tuberculosis disease. For example, among men with body wasting as measured by low BMI, average energy intake was 8.87 ± 4.35 MJ compared with 5.81 ± 3.66 MJ among women with wasting (p<0.05, Table 5). Similarly, among men with moderate-to-severe pulmonary disease as measured by clinical TBscore, average energy intake was 7.00 ± 2.80 MJ compared with 5.17 ± 3.04 MJ among women with moderate-to-severe disease (p<0.05).

**Table 5:** Average 24-Hour Nutrient Intake among Patients with Wasting and Severe Disease Stratified by Sex

**Characteristic [mean, (SD)]**^**c**^	**Wasted**				**Pulmonary tuberculosis disease**			
	**BMI**		**Lean tissue mass**		**Mild**		**Moderate-severe**	
	**Women (n=17)**	**Men (n=18)**	**Women (n=2)**	**Men (n=18)**	**Women (n=16)**	**Men (n=8)**	**Women (n=19)**	**Men (n=20)**
Energy (MJ)	5.81 (3.66)	8.87 (4.35)^b^	2.37 (0.44)	8.54 (4.29)^b^	7.49 (3.12)	12.83 (2.88)^a^	5.17 (3.04)	7.00 (2.80)^b^
Protein (g)	40.5 (25.6)	60.9 (44)	14.3 (1.8)	58.9 (44.5)^b^	57.5 (46.5)	96.6 (43.6)^b^	37.8 (23.7)	42.0 (17.7)
Total fat (g)	30.8 (41.0)	68.6 (53.6)^a^	8.7 (2.6)	63.2 (54.5)	45.4 (40.6)	94.0 (54.6)^b^	27.0 (24.1)	43.1 (34.6)
Carbohydrate (g)	248 (130)	316 (133)	112 (23)	310 (129)^b^	297 (108)	450 (114)^b^	218 (120)	284 (130)
Calcium (mg)	269 (361)	620 (1175)	105 (90)	616 (1177)	656 (1137)	1002 (1718)	259 (303)	356 (381)
Magnesium (mg)	208 (243)	300 (238)	30 (17)	306 (240)^b^	238 (195)	460 (280)^b^	218 (233)	221 (105)
Zinc (mg)	5.3 (4.7)	7.6 (5.2)	1.1 (0.5)	7.4 (5.2)^b^	5.1 (3.2)	11.5 (5.3)^b^	5.1 (4.3)	5.5 (2.2)
Iron (mg)	11.1 (12.5)	12.2 (11.6)	2.0 (0.3)	11.9 (11.6)^b^	10.1 (7.3)	17.5 (15.2)	10.8 (12.5)	9.6 (5.6)
Vitamin A (RE)	432 (382)	591 (833)	99 (40)	566 (839)	774 (756)	704 (930)	397 (428)	444 (664)
Folate (μg)	341 (234)	336 (281)	155 (148)	314 (279)	442 (281)	471 (304)	252 (169)	272 (192)

p-values obtained with Wilcoxon test; ^a^p-value <0.001, ^b^p-value <0.05. ^c^Characteristic values are means ± standard deviation (SD). BMI = body mass index. BMI wasting = <18.5 kg/m^2^ for men and women; Lean tissue mass wasting = Lean tissue mass index <16.7 kg/m^2^ for men and <14.6 kg/m^2^ for women; Mild tuberculosis disease = TBScore ≤5 and moderate-severe = TBScore >5 [20].

## Discussion

In this cross-sectional study of 131 adult HIV sero-positive and sero-HIV negative adults with/or without active pulmonary tuberculosis from urban Uganda, we aimed to establish the independent effects of tuberculosis and HIV infection, the effects of body wasting and tuberculosis disease severity on nutrient intake. We found that the average 24-hour nutrient intake varied by severity of tuberculosis disease, but not by tuberculosis disease or HIV status nor by the nutritional status. In particular, we found that patients with more advanced pulmonary tuberculosis consumed fewer calories, less protein, and less fat. Further, nutrient intake varied by gender regardless of the nutritional status and disease severity. The average 24-hour nutrient intake differed by severity of clinical tuberculosis disease categories of mild and moderate-severe tuberculosis disease. The average 24-hour nutrient intake was comparable by tuberculosis disease and HIV status, and by severity of nutritional status.

Although the differences found in this cross sectional study preclude statements about cause, the findings suggest that in the face of tuberculosis disease, nutrient intake is reduced among patients with more severe disease regardless of HIV infection. In the absence of tuberculosis, nutrient intake was affected by gender, and not HIV infection. We did not discern any differences in nutrient intake by wasting status, regardless of tuberculosis or HIV infection. To our knowledge, this is the first study to show the effect of tuberculosis disease severity on nutrient intake and the gender differences in nutrient intake among individuals with or without tuberculosis. Regarding effects of tuberculosis on nutrient intake during HIV infection, our results are consistent with findings from India in which one study [21] found comparable nutrient dietary intakes between HIV positive patients with tuberculosis and HIV positive individuals without tuberculosis. This Indian study, however, had no comparable group of HIV negative patients with tuberculosis to establish whether there is a synergist effect of tuberculosis and HIV infection on nutrient intake. The strength of our study hinges on the full panel of HIV seropositive and HIV seronegative adults with or without tuberculosis.

The present study demonstrated that disease severity influence nutrient intake at the time of diagnosis but not the mere presence of tuberculosis disease or HIV infection. Minimal differences are present in nutrient intake between tuberculosis patients with wasting and patients without wasting. This suggests that wasting is not a determinant of nutrient intake but is a result of the tuberculosis disease process and a sign of poor intake. During tuberculosis disease, the poor nutrient intake or poor appetite is probably determined by the inflammatory responses to tuberculosis (including release of tumor necrosis factor-alpha) [22] and its interaction with the metabolic pathways that lead to anorexia. Therefore, patients with moderate-severe clinical disease present with marked low appetite that impends nutrient intake other than barriers to food access.

One can postulate that tuberculosis disease affects appetite level equally in men and in women regardless of body composition, leading to compromise of nutrient intake at nearly the same rate regardless of food access barriers. Thus, the gender differences in body composition during tuberculosis as revealed in the present and in previous reports [19] could be explained by the differences in altered metabolism. Men generally, experience higher metabolic rate because of the larger quantities of lean tissue mass compared to women [23] and during tuberculosis, this process is probably pronounced with associated wasting.

The pure gender differences in magnitude of nutrient intakes could be explained by the cultural factors that may compromise intake among women. For example, unequal distribution of food within households [24,25], or men may have the opportunity to eat a wider variety or better quality foods outside the home, such as at local restaurants compared women [26]. The unequal distribution of food within households result from several factors such as women may be trained to show restraint in eating, to give the best foods to men, or to allow others in the family to eat first [27-29].

The major weakness in this study is that our findings were based on the last 24 hours dietary nutrient recall which was not validated by reliable methods such as weighted food record and nitrogen balance studies or measurement of biomarkers such as serum micronutrients. However, we took care to standardize estimation of portion size for actual intake using food photographs and volumetric vessels known by the local population.

## Conclusion

Despite the limitations, the present study revealed that nutrient intake at the time of diagnosis was influenced by tuberculosis disease severity and gender, but not tuberculosis disease or HIV status. Nutritional counseling and supplementation may improve tuberculosis treatment outcomes, but may need to be tailored to severity of disease at presentation and gender.

## Abbreviations

AIDS: Acquired Immune Deficiency Syndrome; BIA: Bioelectrical Impendance Analysis; BMI: Body Mass Index; FMI: Fat Mass Index; HIV: Human Immuno-deficiency Virus; IDI: Infectious Disease Institute; LMI: Lean Tissue Mass Index; MUAC: Mid-upper-arm Circumference; MJ: Mega Joules; NTLP: National Tuberculosis and Leprosy Program; WHO: World Health Organization.

## Competing interests

Mupere E, Parraga MI, Tisch JD, Mayanja KH, and Whalen CC reported no conflict of interest.

## Authors’ contributions

EM, IMP, DJT, HKM and CCW designed research; EM and CCW conducted research and analyzed data; EM, IMP, DJT, HMK and CCW wrote the paper; and EM and CCW had primary responsibility for final content. All authors read and approved the final manuscript.

## Pre-publication history

The pre-publication history for this paper can be accessed here:

http://www.biomedcentral.com/1471-2458/12/1050/prepub
